# The Biocompatibility and the Effect of Titanium and PEKK on the Osseointegration of Customized Facial Implants

**DOI:** 10.3390/ma17174435

**Published:** 2024-09-09

**Authors:** Sung-Ok Hong, Ju-Yeon Pyo, Sung-Woon On, Ja-Yeong Seo, Jin-Young Choi

**Affiliations:** 1 Department of Oral and Maxillofacial Surgery, Kyung Hee University Dental Hospital at Gangdong, Seoul 05278, Republic of Korea; catherine.so.hong.sleepdoc@gmail.com; 2 Department of Pathology, Catholic Kwandong University, International St. Mary’s Hospital, Simgok-ro 100 Gil 25, Incheon 22711, Republic of Korea; 3775140@hanmail.net; 3 Division of Oral and Maxillofacial Surgery, Department of Dentistry, Hallym University Dongtan Sacred Heart Hospital, 7 Keunjaebong-gil, Hwaseong-si 18450, Republic of Korea; drummer0908@hanmail.net; 4 Department of Pathology, SD Lab, 53-21, Dongbaekjungang-ro, Gilheung-gu, Yonging-si 17013, Republic of Korea; milk_sjy@naver.com; 5 Department of Oral and Maxillofacial Surgery, School of Dentistry, Dental Research Institute, Seoul National University, Daehak-Ro #101, Chongno-gu, Seoul 03080, Republic of Korea

**Keywords:** customized facial implant, mandible, bone defect, osseointegration, titanium, implant, SLA, PEKK, biocompatibility, surface modification, CAD/CAM

## Abstract

The purpose of this study was to investigate the optimization of computer-aided design/computer-aided manufacturing (CAD/CAM) patient-specific implants for mandibular facial bone defects and compare the biocompatibility and osseointegration of machined titanium (Ma), Sandblasted/Large-grit/Acid-etched (SLA) titanium, and polyetherketoneketone (PEKK) facial implants. We hypothesized that the facial implants made of SLA titanium had superior osseointegration when applied to the gonial angle defect and prevented the senile atrophy of the bone. Histologic findings of the soft-tissue reaction, hard-tissue reaction, and bone–implant contact (BIC (%) of 24 Ma, SLA, and PEKK facial implants at 8 and 12 weeks were investigated. There was no statistical difference in the soft tissue reaction. Bone was formed below the periosteum in all facial implants at 12 weeks and the BIC values were significantly different at both 8 and 12 weeks (*p* < 0.05). Ma, SLA, and PEKK facial implants are biocompatible with osseointegration properties. SLA can enhance osseointegration and provoke minimal soft tissue reactions, making them the most suitable choice. They provide an excellent environment for bone regeneration and, over the long term, may prevent atrophy caused by an aging mandible. The bone formation between the lateral surface of the facial implant and periosteum may assist in osseointegration and stabilization.

## 1. Introduction 

Iatrogenic, congenital, traumatic, pathological, and age-related degenerative changes can lead to a disfigurement of the craniofacial complex. Facial implants aid in restoration of the facial skeleton such as the mandible, maxilla, chin, malar eminence, nasal dorsum, forehead, and orbital structure [1,2]. While traditionally autogenous bone and cartilage have been used for facial augmentation, their limited availability, donor-site morbidity, increased surgery time, and complications have made them less favorable options in certain cases [3]. With the development of alloplastic biomaterials, complications such as donor-site morbidity can be avoided and surgical time reduced [4]. However, alloplastic materials may also exhibit problems such as wound dehiscence, hematoma, post-operative infection, inflammation, graft explantation, and migration [1]. Thus, an ideal implant should minimize inflammatory cell reactions and maximize stability. 

One method to increase stability is through enhancing the mechanical fitting of the facial implant by using techniques such as computer-aided design/computer-aided manufacturing (CAD/CAM). CAD/CAM technology enables accurate virtual planning, designing, and fabrication, and has great potential for new treatment options such as patient-specific medical devices [5,6]. Customized or patient-specific implants can be used in simple cases to correct asymmetry after esthetic angle reduction or in relatively complex cases to reconstruct the maxillofacial complex after broad tumor excision or panfacial fracture. Currently, the most popular materials are titanium, polyetheretherketone (PEEK), and polyetherketoneketone (PEKK) [7,8].

The second way of increasing stability involves decreasing the mobility of the fitted implant by incorporating bone or enhancing screw fixation. In the 1950s, Brånemark discovered that titanium could be permanently incorporated with bone in a process known as “osseointegration” [9]. The ideal bioactive surface coating should be porous, rough, and have higher surface energy, hydrophilicity, and crystallinity, thus guaranteeing long-term osseointegration [10,11,12]. Titanium has the capacity for osseointegration, and sandblasting, large grit, and acid etching (SLA) process is a safe and predictable procedure that increases implant roughness and enhances cellular adhesion and proliferation [13]. 

Another important prerequisite is the suppression of the soft tissue response through minimizing the graft reaction [14]. The rapid ingrowth of blood vessels and a low inflammatory response are crucial for the successful long-term integration of biomaterials [15,16]. Biomaterial and host reactions are controlled mostly by macrophages, which participate in tissue regeneration or induce extracellular biodegradation, leading to implant failure [17]. Observing the inflammatory reaction after the implantation of different biomaterials is important because identifying an immune-compatible material would guarantee minimal bone resorption due to graft reaction and long-term biocompatibility. 

The objective of this study was to investigate the optimization of CAD/CAM patient-specific implants for mandibular defects using CT imaging and virtual planning and to compare the osseointegration as well as the inflammatory cell response of machined titanium (Ma), SLA titanium, and PEKK facial implants. 

Some of the most widely used alloplastic materials are 1. titanium, 2. polydimethylsiloxane, 3. porous high-density polyethylene (pHDPE), 4. expanded polytetrafluoroethylene (ePTFE), 5. polymethylmethacrylate (PMMA), 6. polyester, 7. polyamide, 8. polyacrylamide, 9. polyalkylimide, 10. hydroxyapatite, 11. hyaluronic acid, 12. collagen, 13. P Poly-L-lactate, and 14. polyaryletherketone (PAEK). Titanium, ePTFE (Gore-Tex, Flagstaff, AZ), polydimethylsiloxane (silicone), pHDPE (Medpor, College Park, GA), PMMA, and PAEK are frequently used as solid implants. ePTFE and polydimethylsiloxane are pliable implants usually applied to small defects that are supported by bone, because larger shapes may cause micromovement and harm adjacent soft and hard tissue. Both pHDPE and PMMA can be applied to larger defects, but they lack osseointegration properties and can cause bone resorption below the implant and thinning of the overlying skin [18].

Titanium and its alloys (mainly Ti-6Al-4V) have high biocompatibility and exert exceptional corrosion resistance, making them the metals of choice [19]. The stoichiometric composition of commercially pure titanium allows its classification into four grades that vary mainly in their oxygen content, with grade 4 having the most (0.4%) and grade 1 the least (0.18%) [20]. Titanium implants can attract neighboring proteins, cells, and body fluids because of their metallic properties (valence charge). Bioactive surface modifications seem to improve the performance of dental implants, as proven by histomorphometry (BIC, peri-implant bone density) and biomechanical testing (removal torque, push-out/pull-out test, and resonance frequency analysis) [21]. Surface roughness and porosity enhance osteoblast-like cell adhesion and affect the configuration and conformation of cellular pseudopodi, which aids in cell adhesion [22,23]. SLA titanium is better able to achieve osseointegration, has a significantly higher removal torque, and less coronal bone loss; therefore, it is currently the most commonly used material for dental implants [24]. 

PAEK is a family of high-temperature thermoplastic polymers with mechanical properties that can coexist with human bone. PEKK and PEEK are both members of the aryl ether ketone family of polymers. PEEK is nonallergenic, nonmagnetic, radiographically translucent without any artifacts, and does not produce exothermic reactions like PMMA [25]. The elasticity of PEEK is comparable to that of cortical bone and can provide permanent long-term results that can also be sterilized repeatedly without affecting its composition or mechanical stability. However, a disadvantage of pure PEEK is that it has no bioactive potential [26]. 

PEKK is a high-performance methacrylate-free thermoplastic material introduced by Bonner in 1962 [26]. PEKK filaments are semi-crystalline polymers with a density of 1.4 g/cm^3^ and a yield strength of 115 MPa. The modulus of elasticity of PEKK (3–4 GPa) is similar to that of bone (18 GPa), which provides a resolution to the stress shielding problems incurred with implants such as metal (100–210 GPa), thus minimizing implant failure [17]. The presence of the second ketone group leads to more SO_3_H on the surface of PEKK than in the case of PEEK [26]. This makes the surface topography more complex and displays both amorphous and crystalline behavior and increases the surface area and the micro-roughness, ultimately enhancing the cell behavior and osseointegration on the surface of PEKK [26]. PEKK has a higher thermostability, elevated mechanical strength, and more ketone groups than PEEK. This allows for diverse surface chemical modifications that can increase its bioactivity [25]. PEKK is an emerging biomaterial that has been the subject of fewer studies than its counterpart PAEK family, but an increased number of applications in dental prostheses, dental implants, and facial implants is anticipated.

## 2. Materials and Methods

Three distinct types of maxillofacial implant surfaces were investigated in our study: Ma titanium, SLA surface titanium, and PEKK.

### 2.1. Preparation of Samples

Titanium specimen preparation

The titanium specimens used for the in vitro and in vivo tests were divided into two groups: Ma and SLA. Ma and SLA commercially pure grade-4 titanium implants (Megagen, Daegu, Republic of Korea) were manufactured by the traditional subtractive method using a computer numerically controlled (CNC) machine (ZX-5SM, MANIX, Seoul, Republic of Korea). Ma, SLA titanium implants were ultrasonicated and dried at room temperature. The SLA titanium specimens were sandblasted with Al_2_O_3_ particles and etched using a mixture of HCl and H_2_SO_4_.

PEKK specimen preparation

PEKK implants were machined out of commercially available medical-grade PEKK reinforced with titanium dioxide (TiO_2_) powder. (Pekkton^®^ ivory, Cendres + Métaux Holding SA, Biel/Bienne, Switzerland). PEKK implants had a smooth finish and a surface roughness attributable to the CNC milling process.

### 2.2. MA, SLA, and PEKK Implant Surface Analyses

Surface topography, surface roughness, and chemical component analysis were performed on the Ma titanium implants, the SLA implants, and the PEKK surfaces. Qualitative surface roughness analyses of the implants were conducted using a field-emission scanning electron microscope (FE-SEM; SU5000, Hitachi High-Tech Corporation, Tokyo, Japan). The surfaces were randomly observed at different sites at different magnifications in secondary electrons (SE) with working distance of 12.2–12.9 mm and an operational voltage of 15.0 kV. The elemental composition of each implant was checked using Energy Dispersive X-ray Spectroscopy (EDS; Xplore 30, Oxford Instruments, Abingdon, UK). Quantitative surface roughness was evaluated using a 3D laser scanning microscope (LEXT™ OLS5100-SAF, Olympus, Tokyo, Japan) with an analysis area of 1024 × 1024 μm.

### 2.3. Virtual Surgical Planning and Computer-Aided Implant Design/Fabrication

**Figure 1:**
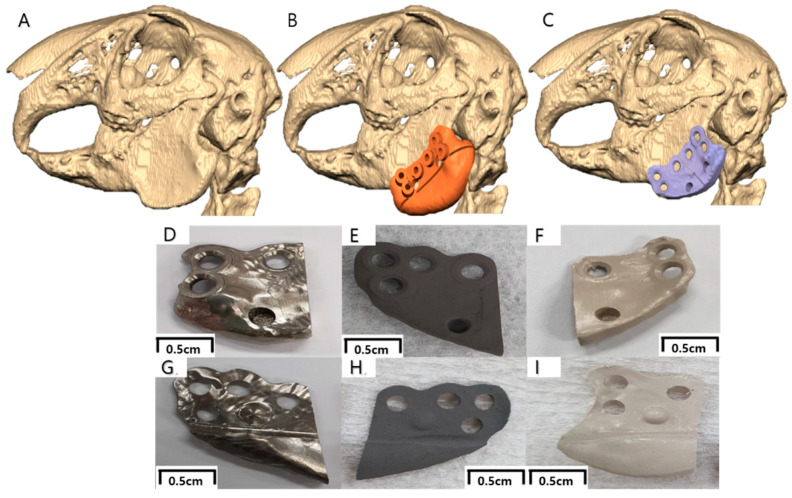
Three-dimensional reconstruction of the maxillomandibular complex and customized final implant of a rabbit using CAD/CAM technology. (**A**) Three-dimensional STL conversion, (**B**) Surgical guide for the left mandible angle, (**C**) Customized final implant design on the left mandible angle, (**D**) Customized Ma implant lateral side (soft tissue contact side) with indentation, (**E**) Customized SLA implant lateral side with indentation, (**F**) Customized PEKK implant lateral side with indentation, (**G**) Customized MA implant medial side (hard tissue contact side) with protrusion, (**H**) Customized SLA implant medial side with protrusion, (**I**) Customized PEKK implant medial side with protrusion. (Abbreviations: STL, stereolithography; CAD/CAM, computer-aided design/computer-aided manufacturing; Ma, machined titanium; SLA, sandblasted, large grit, acid-etched titanium; PEKK, polyetherketoneketone).

Digital imaging and communications in medicine (DICOM) files were extracted from the CT scan and converted to 3-dimensional (3D) stereolithography (STL) files, which were planned and manufactured in collaboration with a surgeon and an engineer for 3D modeling and computer-aided design/computer-aided manufacturing (CAD/CAM; Geomagic Freeform Plus, 3D systems, San Diego, CA, USA). During the virtual planning, a surgical guide was made to cut the angle of the mandible without affecting the surrounding inferior alveolar nerve, tooth roots, and other critical anatomical structures. The guide was also designed to place three screws in anatomically stable positions on the mandible, and a hole for guide reference. The guide was produced using a liquid-crystal planar solidification (LCPS) 3D printing system (RAM500, Ray, Yongin-si, Republic of Korea). A customized 3D facial implant replicating the original mandible shape was designed to accurately fit the end of each mandible angle and was produced using a milling machine (ZX-5SM, MANIX, Seoul, Republic of Korea) (Figure 1). Three screw fixation holes were made in the implant, and two implants were designed to fit on each side of the mandible. An indentation on the lateral side of the implant was designed as a reference point for soft tissue inflammation, and a protrusion was made on the lingual side of the implant as a reference point for bone evaluation (Figure 1).

### 2.4. In Vivo Studies

Approval for animal experiment

All the animals were approved by the Animal Care and Use Committee of the Seoul National University School of Dentistry, Korea. (IRB number: SNU-200717-1-1). All methods were performed following relevant guidelines and regulations. The study was carried out in compliance with the ARRIVE guideline.

Animals and CT taking

Six healthy female New Zealand white rabbits weighing 3–3.5 kg were used in this study. All the animals were housed in a temperature-controlled room with a 12 h alternating light–dark cycle and were given water and food throughout the study. All the animals were acclimatized to their surroundings for at least two weeks before the study.

Pre-operative multi-slice computed tomography (Revolution Frontier™ CT, GE Healthcare, Seoul, Republic of Korea) with a 0° gantry tilt was obtained from each rabbit with a slice thickness of 1 mm under general anesthesia using an intramuscular injection of xylazine HCl (Rompun^®^, 10 mg/kg, Bayer Korea, Seoul, Republic of Korea) and ketamine HCl (Ketalar^®^, 50 mg/kg, Yuhan, Seoul, Republic of Korea).

Surgical procedure

**Figure 2:**
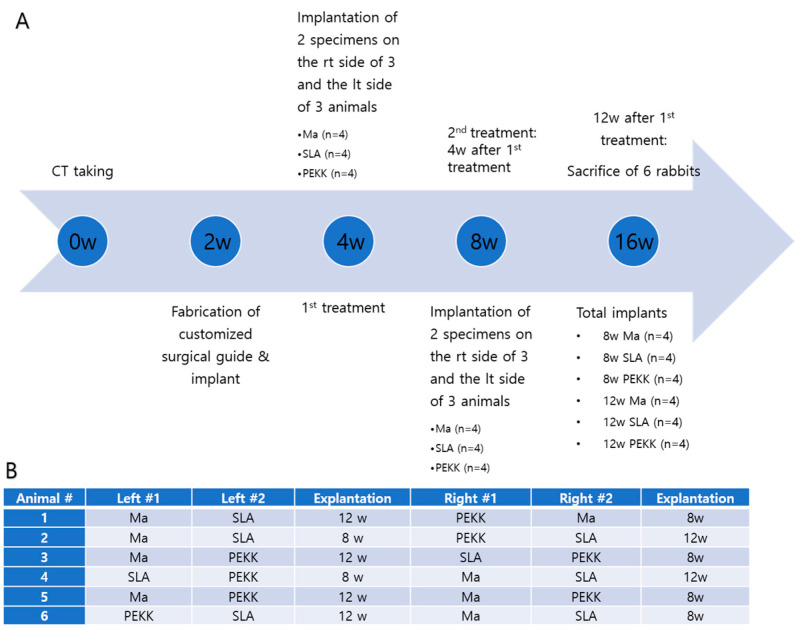
(**A**) Schematic timeline of the animal experiment, (**B**) Table showing randomization of the animal experiment. Twenty-four implants were randomized into six rabbits. Ma, SLA, and PEKK implants were randomized on the right and left mandibles so that each rabbit had four implant specimens that were sacrificed to include two 8 w (*n* = 12) and two 12 w (*n* = 12) implants. (Abbreviations: w, week; rt, right; lt, left).

The specimens were randomly divided into six groups (*n* = 4 in each group). In each rabbit, four implants were randomly inserted into both mandibles. Surgery was implemented on both sides of the rabbits, with two implants inserted on one side of the rabbit mandible. To minimize complications, the surgical time did not exceed 2.5 h; therefore, implantation on each side was spaced four weeks apart. A total of 24 implants (Ma, SLA, or PEKK) were inserted into the mandibles of six rabbits. The implants were randomized according to Figure 2.

All the surgical procedures were performed under general anesthesia. General anesthesia was obtained after intramuscular injection of xylazine HCl and ketamine HCl, and a booster injection of 2/3 of the initial amount was administered 1 h after the initial injection. Antibiotic prophylaxis was administered 30 min before incision with an intramuscular injection of amoxicillin–clavulanate (Clamoxin^®^, 30 mg/kg, Shin Poong Pharm, Seoul, Republic of Korea). Under a sterile condition, a 5 cm incision was made on the submandibular level of the skin, and dissected until the mandibular ramus was exposed. After placing the surgical guide on the mandible angle, a 2.0 mm drill bit was used to produce three identical holes for screw placement, and a 2.3 mm round bur was used to drill a hole to encompass the indentation. A disk was used to cut the mandibular angle.

**Figure 3:**
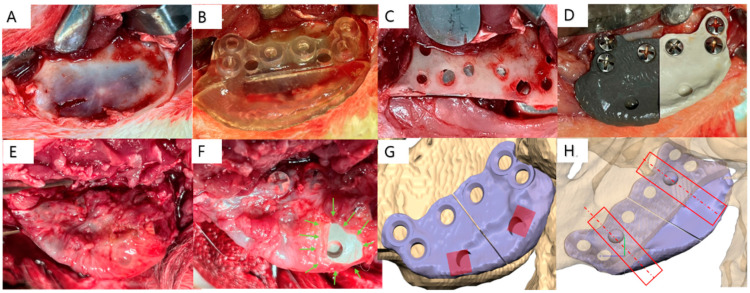
Peri-operative photographs of angle reduction and facial implant insertion in a rabbit. (**A**) Exposed mandible angle after skin flap dissection, (**B**) A customized surgical guide was placed on the mandibular angle, (**C**) Screw holes and reference holes were made using a surgical guide for the insertion of two implants, (**D**) SLA and PEKK implants were inserted and fixed with screws—post-operative photograph and illustration of the left angle, (**E**) Exposed angle with overlying periosteum at 12-weeks after implantation, (**F**) Fixed SLA and PEKK implants with excised lateral periosteum. The green arrows show the excised tissue, (**G**) The location of the soft tissue sample. The red shaded box represents the excised area, (**H**) Location of the bone tissue sample. The specimen’s cross-section passes through the protrusion on the medial side of the implant. The plane of the cross-section is shown as a dotted line, and the red open box is the sample boundary.

All the metal and PEKK implants were autoclaved before the surgery. The adaptability of the implant was checked and screwed with three 2.4 mm diameter, 6 mm length maxi screws (Jeil Medical, Seoul, Republic of Korea). In areas with a thin cortical lining, 2.6 mm diameter and 6 mm length maxi screws (Jeil Medical, Seoul, Republic of Korea) were used. The muscle, subcutaneous tissue, and skin were sutured in layers with resorbable sutures (Vicryl 3-0, Ethicon, Somerville, NJ, USA) and an intramuscular injection of diazepam (1 mg/kg, Samjin Pharm, Seoul, Republic of Korea) was administered. The same surgical procedure was performed on the contralateral side, 4 weeks later (Figure 3).

Animal sacrifice and retrieval of specimens

The animals were sacrificed (CO_2_ euthanasia chamber) after sedation with ketamine, xylazine, and diazepam after healing periods of 8 and 12 weeks [21,27,28]. The soft tissue adjacent to the lateral surface of the facial implant was excised, and the indentation in the implant was used as a reference point. The jaws were dissected, and blocks containing the experimental specimens were obtained (Figure 3).

### 2.5. Histological Preparation and Analysis

Histological preparation

Hematoxylin and Eosin (H&E) slides of soft tissue specimens at 8 and 12 weeks were prepared. The whole excised soft tissue was cut into approximately 3 µm slices resulting in two to three sections per specimen, fixed onto one slide.

The mandibles containing the implants that were harvested at 8 and 12 weeks were embedded in light-curing epoxy resin (Technovit 7200VLC, Heraeus Kulzer, Dormagen, Germany) without calcification. The specimen was then cut along the long axis using an EXAKT diamond cutting system (EXAKT 300 CP, Oklahoma City, OK, USA) around the protrusion on the medial side of the implant. The slide was ground to a thickness of 45 ± 5 µm using an EXAKT grinding system (EXAKT 400CS, Oklahoma City, OK, USA) and then stained with Goldner trichrome staining.

Histological analysis

Histologic sections were analyzed by two experienced pathologists who were blinded to the study’s purpose. To evaluate the soft tissue reaction lateral to the implant, H&E sections were evaluated using a light microscope (BX53, Olympus Corporation, Tokyo, Japan) and an image analysis software (CaseViewer^®^ ver.2.0, 3D Histech, Budapest, Hungary). The inflammatory reaction of the adjacent tissue on the lateral side of the implant was determined by assessing the number of inflammatory cells (plasma cells, macrophages, neutrophils, eosinophils, basophils, and T lymphocytes) in the tissue sections, according to the following: 1. The percentage (%) of inflammatory cell infiltration in the whole tissue of the specimen; 2. The inflammation field severity (minimal/mild/moderate/severe); 3. Granulation tissue formation (absent vs. present); 4. Pattern of fibrosis (score: 0–3).

**Figure 4:**
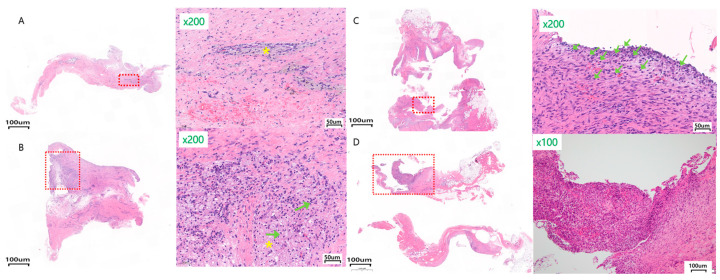
Representative histological images (H&E staining) of various inflammatory percentages (%) of the whole biopsy specimen in different implants. (**A**) At 5% inflammation: 8 w SLA at a magnification of ×17 & ×200 showing foamy histiocyte, (**B**) At 15% inflammation: 12 w SLA at a magnification of ×16 & ×200 showing foamy histiocytes and lymphoplasma cells, (**C**) At 30% inflammation: 8 w Ma at a magnification of ×10 & ×200 showing lymphoplasma cells, (**D**) At 50% inflammation: 12 w Ma at a magnification of ×15 and ×100 showing granulation tissue formation. The yellow * represents foamy histiocytes and the green arrows represent lymphocytes.

The percentage of inflammatory cell infiltration in all two to three paraffinized sections that comprised the whole specimen was recorded (Figure 4).

**Figure 5:**
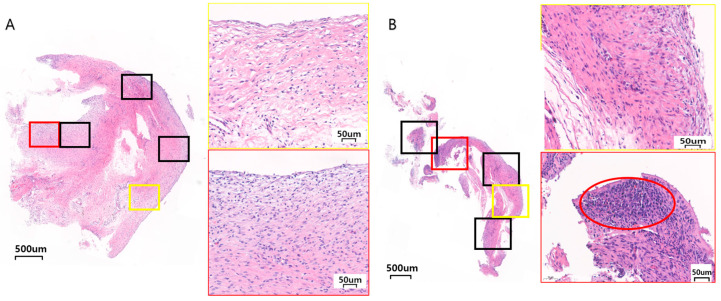
Representative histological images (H&E staining) of various inflammatory field severity (minimal, mild, moderate, severe) in two (yellow and red box) of five sites of one specimen at magnifications of ×200. The black boxes are the other three sites that were counted to find the inflammatory field severity. (**A**) Image of 12 w Ma showing minimal inflammation in the red box, and minimal inflammation in the yellow box, (**B**) Image of 8 w SLA showing mild inflammation in the yellow box, and severe inflammation in the red box. The red circle shows aggregates of inflammatory cells.

The inflammation field severity was checked in five 10 × 10 grid areas (0.25 mm^2^) that had the largest aggregations of inflammatory cells at a magnification of 200 times the original size. The average number of mononuclear inflammatory cells in the five areas was checked and scored from 0 to 3 (0: absent to minimal, <10%; 1: mild, 10–30%; 2: moderate, 30–60%; 3: severe, >60%) (Figure 5) [29].

**Figure 6:**
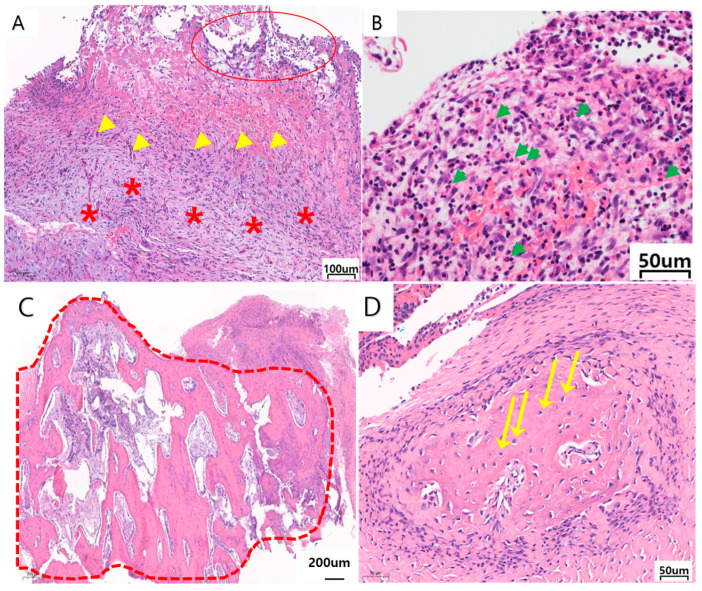
Representative histological images (H&E staining) of soft tissue present in 12 w implants. (**A**) PEKK medial surface at a magnification of ×100 showing granulation tissue formation composed of necro-inflammatory exudates, capillary proliferation, and loose myxoid fibrosis. The red * represents myxoid fibrosis, the yellow arrows represent multinucleated giant cells, and the red circle represents exudates, (**B**) Granulation tissue of Ma medial surface at a magnification of ×400. The green arrows represent neutrophils, (**C**) Bone formation on the PEKK lateral surface at a magnification of ×40. The dotted red lines show areas of bone formation, (**D**) Woven bone formation, and osteocytes on the PEKK lateral surface at a magnification of ×200. The yellow arrow shows osteocytes.

For granulation, a combination of capillary endothelial cells, fibrosis, and inflammatory cells was inspected. (Figure 6).

**Figure 7:**
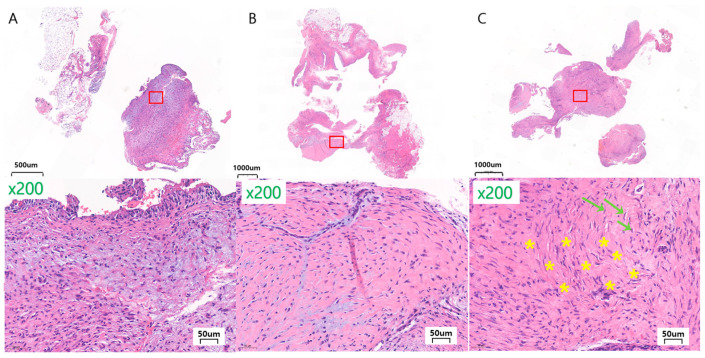
Representative histologic images (H&E staining) scoring the different degrees of fibrosis (1–3). (**A**) 1: loose myxoid fibrosis on 12 w PEKK, (**B**) 2: focal thick fibrosis on 8 w Ma, (**C**) 3: diffuse thick fibrosis on 8 w Ma. The yellow * represents collagen bundles and the green arrows represent intermingled fibroblasts.

For fibrosis, the presence of fibroblasts and collagen was investigated and scored from 0 to 3 depending on the amount and pattern (0: absent, 1: loose, thin fiber, 2: thick collagen, focal, 3: thick collagen, diffuse) (Figure 7).

To evaluate and quantify the response of the bone surrounding the implants, histological analyses were performed using digital images (Pannoramic 250 Flash III, 3D Histech, Budapest, Hungary) and image analysis software (CaseViewer^®^ ver.2.0, 3D Histech, Budapest, Hungary). The new bone formation between the lateral surface of the facial implant and the periosteum was evaluated and marked as absent or present.

Osseointegration between the mandible and medial surface of the facial implant was measured. The bone–implant contact (BIC) was analyzed in the selected sections where a hole in the bone was formed to position the protrusion of the lingual surface of the implant. The bone attachment from below this hole to the area where the cortical bone was cut was analyzed. The BIC was quantified in four implants for each type of implant surface at 8 and 12 weeks, totaling 24 specimens. The BIC was determined using the following formula:

$$BIC\left(\%\right)=\frac{sum\;of\;the\;length\;of\;BIC}{circumference\;of\;select\;implant\;regions}\times 100$$

BIC was defined as the interface where the bone tissue was located within 20 µm of the implant surface without any intervention of the soft tissue.

### 2.6. Statistical Analysis

The data were analyzed using the SPSS Statistics 25 software (IBM Corporation, New York, NY, USA). Fisher’s exact test was used to check the relationship between the type of facial implant and soft tissue variables. The McNemar test (EXACT) was used to compare the soft tissue variables at 8 and 12 weeks. An independent *t*-test and one-way ANOVA were used to compare the three facial implants, inflammation percentage, surface roughness, and BIC. The data were reported using the mean, standard deviation (SD), range, 95% confidence interval (CI), and median. A Bonferroni correction for the alpha errors was used, and the results were considered statistically significant at a *p* value of <0.05. Inter-observer reliability was calculated using the intraclass correlation coefficients (ICC) and Cohen’s kappa.

## 3. Results

### 3.1. Implant Surface Analyses

**Figure 8:**
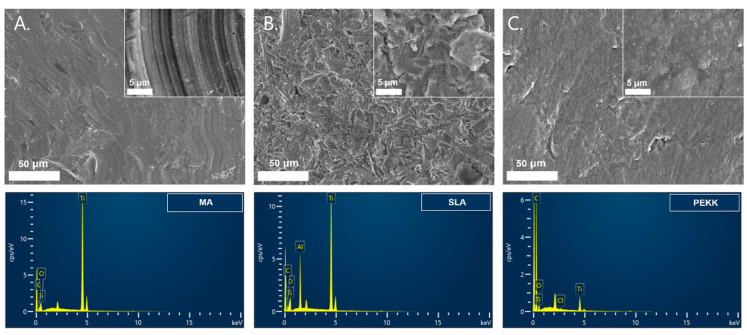
Surface morphology and component analysis using FE-SEM and EDS. SEM magnifications were at high power (×5.00k) and low power (×500). (**A**) Ma surface. Particle elements of Ti, C, and O exist. (**B**) SLA surface. Particle elements of Ti, O, Al, C exist. (**C**) PEKK surface. Particle elements C, O, Ti, and Cl exist. (Abbreviations: FE-SEM, field emission scanning electron microscopy; EDS, energy dispersive X-ray spectroscopy).

Under FE-SEM, Ma surfaces had the characteristic parallel machining grooves produced during manufacturing. High-power magnification of the autoclaved SLA surface implants revealed irregularities, micropits, and surface roughness with alumina particles. PEKK surfaces had slightly rough surfaces (Figure 8).

Analysis of the chemical composition of the pure commercial titanium revealed minimum impurities, such that the criteria for commercially pure grade-4 titanium were satisfied. The elemental composition of the commercially pure grade-4 titanium used in this study included carbon (0.051%), iron (0.14%), oxygen (0.36%), nitrogen (0.001%), yttrium (< 0.0004), and titanium (balanced). After manufacturing the 3D implant, Ma surfaces had particle elements of Ti 85.9%, O 7.53%, and C 6.54%. SLA surfaces had particle elements of Ti 60.6%, O 23.3%, Al 9%, C 6.9%, and PEKK had elements C 72.7%, O 17.5%, Ti 9.5%, and Cl 0.25% (Figure 8).

**Figure 9:**
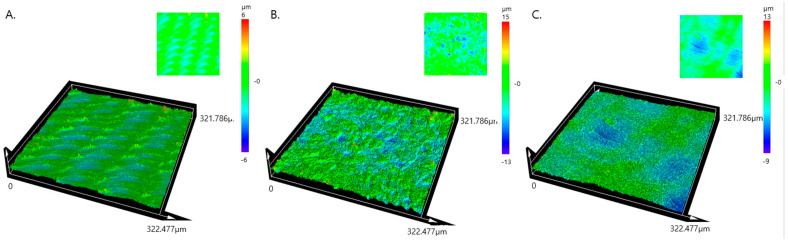
Microscope image showing surface roughness (Sa) of (**A**) Ma is minimally rough, (**B**) SLA is rough, (**C**) PEKK is moderately rough.

The average surface roughness (Sa, arithmetical mean height) of Ma was 0.871 ± 0.07 μm, SLA 2.174 ± 0.07 μm, and PEKK 1.451 ± 0.06 μm, with the surface roughness between Ma, PEKK, and SLA being statistically significant (*p* < 0.05). (Figure 9).

### 3.2. In Vivo Histological Observations and Analyses

General finding

The post-operative healing was uneventful in all the rabbits. No complications, such as allergic reactions, abscesses, or infections, were observed throughout the study. One rabbit experienced sialocele post-operatively on one side, but this was relieved with drainage and compression and one week of antibiotics (amoxicillin, IM 30 mg/kg).

Assessment of soft tissue reaction

The inter-observer ICC was calculated to evaluate the reliability of the measurements. All the measurements exhibited excellent inter-observer reliability ranging from 0.75 to 1.00, which was statistically significant.

Inflammation percentage

The average inflammation percentages for Ma, SLA, and PEKK at 8 weeks were 20.63 ± 10.48%, 14.25 ± 17.44%, and 5.88 ± 3.12%, respectively. At 12 weeks for Ma, SLA, and PEKK, they were 25.63 ± 16.38%, 12.63 ± 6.52%, and 15.75 ± 11.79%, respectively. The PEKK facial implants produced the lowest percentage of inflammation, while the Ma surfaces produced the highest, but there was no statistical significance between Ma, SLA, and PEKK at 8 and 12 weeks (Table 1, Figure 4).

Field severity (minimal, mild, moderate, severe)

The field severity in the Ma implants had one severe, two moderate, and five mild. The SLA implants showed three severe, three mild, and three minimal. The PEKK implants had four mild and four minimal. The SLA implants had the greatest number of severe fields of inflammation, while the PEKK implants had the fewest, but there was no significant difference between Ma, SLA, and PEKK at 8 and 12 weeks (Table 1, Figure 5).

Granulation tissue formation

Granulation formation was observed in two Ma facial implants at 8 and 12 weeks. One PEKK facial implant also exhibited granulation formation at 12 weeks. There was no significant difference between Ma, SLA, and PEKK at 8 and 12 weeks (Table 1, Figure 6).

Fibrosis

Fibrosis was observed in all the specimens. Loose and thin collagen were observed in the three PEKK facial implants. Thick focal collagen was observed for five Ma, three SLA, and three PEKK facial implants. Thick diffuse collagen was noticed with three Ma, five SLA, and two PEKK facial implants. The SLA implants incurred the most severe fibrosis while the PEKK implants exhibited the least, but there was no significant difference between Ma, SLA, and PEKK at 8 and 12 weeks (Table 1, Figure 7).

Other findings

Focal bone formation was found in three specimens (two of the 8-week specimens, one of the 12-week specimens) of soft tissue near PEKK, and calcification was observed in the soft tissue of one of the Ma facial implants at 12 weeks. Hemosiderin-laden macrophages were observed in three soft tissue specimens for the metal implants (one 8-week Ma, one 8-week SLA, one 12-week SLA), and dust-laden macrophages were observed in the soft tissue of one SLA specimen at 8 weeks. Neutrophilic histiocyte aggregates were also observed on one Ma specimen at 12 weeks (Figure 5 and Figure 6).

### 3.3. Assessment of Mandible Defect Repair and Implant Osseointegration

All the specimens exhibited new bone formation or active bone formation on the inner cutting side of the cortical bone and the inner portion of the bone marrow, regardless of the BIC status. Partial implant surfaces were surrounded by newly formed trabeculae of woven bone, but some had fibrosis around the bone. Histologic evidence of osteoblastic activity surrounding the implants and absent foreign body inflammation indicated healthy bone remodeling and biocompatibility.

Mandible defect and implant medial surface

**Figure 10:**
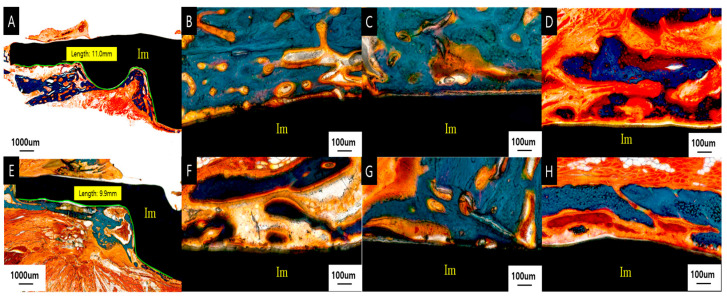
Histologic section stained with Goldner trichome showing the medial surface of the implant at 8 weeks (**A**–**D**) and 12 weeks (**E**–**H**). The green line is the circumference of the implant region where the BIC was measured. (**A**) PEKK surface at a magnification of ×16, (**B**) Ma surface at a magnification of ×100, (**C**) SLA surface at a magnification of ×100, (**D**) PEKK surface at a magnification of ×100, (**E**) PEKK surface at a magnification of ×17, (**F**) Ma surface at a magnification of ×100, (**G**) SLA surface at a magnification of ×100, (**H**) PEKK surface at a magnification of ×100. (Abbreviations: BIC, bone-implant contact; Im, implant).

At 8 weeks after implantation, all of the Ma, SLA, and PEKK implants had woven with the lamellar bone histologically in direct contact with the surrounding medial bone, with no signs of inflammation at the bone–implant interface (Figure 10).

**Figure 11:**
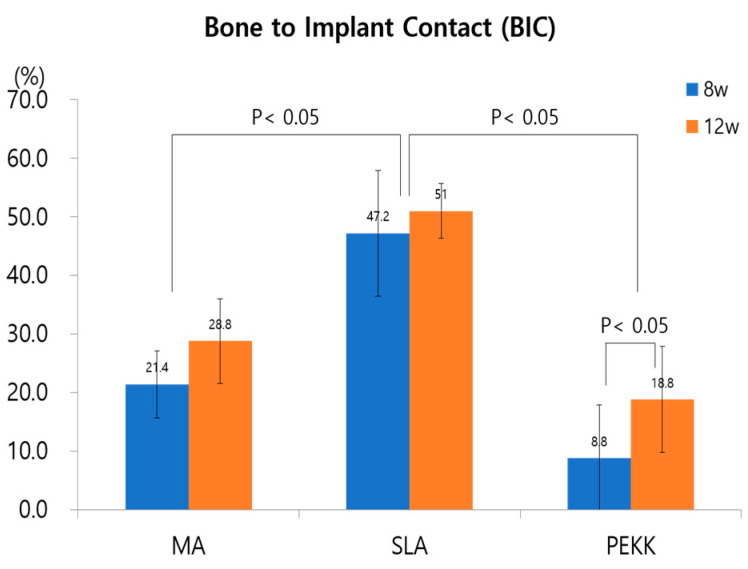
BIC percentages for MA, SLA, and PEKK, 8 and 12 weeks after implantation. The BIC between the Ma, SLA, and PEKK facial implants was statistically different. The BIC between PEKK at 8 and 12 weeks was statistically different.

At 8 weeks, the proportion of direct BIC with the Ma, SLA, and PEKK implants was 21.4 ± 10.7%, 47.2 ± 9.0%, and 8.8 ± 5.7%, respectively. The SLA group had the highest BIC value, which was significantly higher than that of the other two groups (*p* < 0.05). The SLA group had a higher BIC value at 8 weeks than Ma, while the PEKK group had the lowest BIC (Figure 11). At 8 weeks, Ma and SLA exhibited significant differences, and PEKK and SLA also had significant differences in terms of their BIC values (Ma vs. SLA (*p* < 0.05); SLA vs. PEKK (*p* < 0.05)).

Twelve weeks after implantation, all the Ma, SLA, and PEKK implants had some woven and mostly lamellar bone that was histologically in direct contact with the surrounding medial bone, with no signs of inflammation at the bone–implant interface (Figure 10). Twelve weeks after implantation, the BIC values of the Ma, SLA, and PEKK implants were 28.8 ± 4.6%, 51.0 ± 9.0%, and 18.8 ± 7.2%, respectively. The SLA group had the highest BIC value (*p* < 0.05), followed by the Ma and PEKK groups (Figure 11). At 12 weeks, Ma and SLA were significantly different, and PEKK and SLA also exhibited significant differences in terms of their BIC value (Ma vs. SLA (*p* < 0.05); SLA vs. PEKK (*p* < 0.05)).

Comparing the BIC values of the Ma, SLA, and PEKK implants at 8 and 12 weeks revealed that only the differences between the PEKK implants at 8 and 12 weeks were statistically significant (*p* < 0.05). All surfaces were compared regardless of the implantation time. The mean BIC ratio was highest in the SLA surface implants at both 8 and 12 weeks, with significant differences between the three types of surface implants (*p* < 0.05; Figure 11).

Implant lateral surface and periosteum

**Figure 12:**
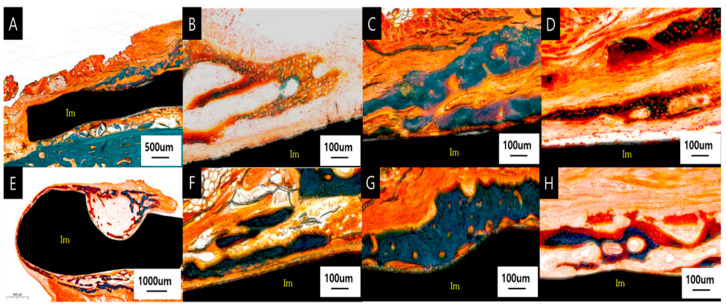
Histologic section stained with Goldner trichrome, showing the lateral surface of the implant at 8 weeks (**A**–**D**) and 12 weeks (**E**–**H**). Woven bone is dark red and green with clear and pinkish specks, lamellar bone is green and turquoise. (**A**) SLA surface at a magnification of ×20, (**B**) Ma surface at a magnification of ×100, (**C**) SLA surface at a magnification of ×100, (**D**) PEKK surface at a magnification of ×100, (**E**) PEKK surface at a magnification of ×10, (**F**) Ma surface at a magnification of ×100, (**G**) SLA surface at a magnification of ×100, (**H**) PEKK surface at a magnification of ×100.

At 8 weeks, new bone formation, mostly encapsulated by fibrous tissue, was observed on the lateral side of the implant below the periosteum, with moderate amounts of woven and lamellar bone. In the SLA group, bone formation was observed on all lateral surfaces of the four facial implants; for the Ma and PEKK groups, bone formation was observed for two of the four implants. (Figure 12) At 12 weeks, moderate amounts of new bone formation partially encapsulated by fibrous tissue were observed on the lateral side of the implant below the periosteum, which had undergone remodeling to become mature lamellar bone. All four of the four Ma, SLA, and PEKK facial implants had some bone formation (Figure 12).

## 4. Discussion

### 4.1. Soft Tissue Reaction on Facial Implants

To avoid excessive tissue inflammation, an ideal alloplastic implant should have excellent biocompatibility, possess the characteristics of the tissue it replaces or augments, be nonallergenic, noncarcinogenic, and nontoxic [2]. The infection rates of facial implants have been the subject of many studies, with rates ranging from 7.7% to 14.3% [7,30]. In this study, there were no cases of implant infection such as the presence of pus, implant extrusion, or removal.

Neutrophils are the most abundant type of leukocytes in the body. Traditionally, these were only thought to play a key role in an acute inflammatory response, but they also play a part in chronic inflammation [31]. Trindade et al. [10] studied titanium implants to demonstrate the involvement of the immune system in osseointegration. Neutrophils were present 4 weeks after the acute inflammation period, attributing their prolonged presence to the relationship between the titanium surfaces and the upregulation of neutrophil cytosolic factor 1 (NCF-1). In our study, neutrophil histiocyte aggregates were also found on the surface of a Ma implant, but this was at 12 weeks. This is much longer than previously reported and may be explained by the suppressive role macrophages have in neutrophil apoptosis and the pro-regenerative neutrophil contribution. Reparative neutrophils can appear at certain time points after injury and may be associated with increased vascularity around the implant since they have been related to aiding the revascularization in transplanted tissue hypoxia and carcinogenesis [31]. Therefore, the appearance of neutrophils in this study does not seem to be a key effector in inflammation, but instead supports the functionality of other cell types, although immunohistochemical staining is needed to interpret the exact etiology.

Macrophages exist in a variety of functional states, distinguished as anti-inflammatory (M2 phenotype) or pro-inflammatory (M1 phenotype). In this study, hemosiderin-laden macrophages and dust-laden macrophages were observed in the soft tissue of the metal implants. Hemosiderin-laden macrophages, pink with digested RBCs, are usually found in organs that have experienced chronic bleeding due to trauma [32]. The presence of this macrophage on metal surfaces may only be because more pressure and friction on the bone are generated after screw fixation with this relatively heavier and rigid facial implant. Dust-laden macrophages, grayish with digested metal particles, are common within the alveoli of the lungs of coal workers, who work in the presence of large quantities of dust [33]. The etiology of these macrophages in this study was thought to be metal particles that were produced by the drilling step. The release of titanium particles and ions has been reported from the instrumentation used for implant preparation, the implant surfaces during insertion, and the abutment interface during insertion and functional loading [34].

Fibrosis is defined by the overgrowth, hardening, and scarring of various tissues attributed to the excess deposition of extracellular matrix components (ECM) [35]. Fibrosis was present in all the soft-tissue specimens with SLA having the thickest and most diffuse collagen, while PEKK had the least. Mechanical injury from procedures such as surgery can trigger an excessive accumulation of ECM, leading to the formation of permanent fibrotic scars [35]. In this study, the thickest diffuse collagen was seen in SLA facial implants, probably because SLA has the fastest rate of cellular infiltration when compared to Ma and PEKK [22]. SLA surfaces modified in dental implants hasten the rate of cellular attachment and differentiation, and protein adsorption or hemocompatibility can also be improved [22].

Granulation tissue is confirmed by existing macrophages, neovascularization (capillary formation), and fibrosis. Granulation tissue may be a precursor to fibrous capsule formation and foreign body reactions. The Ma facial implants were found to have the most granulation tissue, while the SLA had none. One reason may be that etching can have a biomimetic effect and reduce the number of pro-inflammatory M1 macrophage phenotypes [22]. Another reason may be that, in a functioning mandible, the periosteum does not quickly adhere to the smooth machined surface, which causes friction or micromovement of the soft tissue in the initial healing period, adversely affecting the initial stability of the facial implant [36].

The different soft tissue reactions between PEKK, MA, and SLA are the consequence of different rates of cell attachment and growth on these biomaterials. Fibroblasts and osteoblasts adhere differently to PEKK and titanium [37]. Implants with porosity promote tissue ingrowth and increase the chances of bacterial invasion. Solid implants do not allow tissue ingrowth but increase the chances of fibrotic encapsulation or migration [1,38]. SLA surfaces may cause more inflammatory responses because the surfaces with small pores can make soft tissue infiltration easier; therefore, in this study, the field severity and fibrosis were more severe in this group. PEKK had the least amount of granulation tissue, less fibrosis, and the smallest distribution of inflammatory cells, making it the most attractive facial implant concerning soft tissue.

### 4.2. Hard Tissue Reaction on Facial Implants

Ma, SLA, PEKK-surfaced implants, and bone

The surface modification of titanium implants has been suggested as a means of improving osseointegration [39]. Surface modifications can increase the bone tissue response around the implant by stimulating the healing process and forming new bone [40]. Many studies have shown that a rough implant surface, compared to a relatively smooth surface, promotes better protein adsorption, increases extracellular matrix deposition, and improves the differentiation toward osteoblastic cells [41]. SLA titanium increases biocompatibility in the early bone-formation stage, stimulates cell differentiation, and positively affects the activation of blood platelets and cell migration [42]. Many studies have proven that SLA produces a higher BIC value than an oxidized surface, implying that such surfaces have a higher affinity for bone than the oxidized surface during the initial healing period [43].

Both implants exhibited high BIC and osseointegration, but the roughened SLA surfaces showed earlier fixation in the bone tissue and a higher BIC value. The histologic results of this experimental study demonstrate a good bone response with an important formation of new bone around the implant surface after the healing period. The BIC value for the SLA surfaces at 8 and 12 weeks was higher than that for the Ma surfaces. This means that faster osseointegration occurred in the SLA group because of its rough texture, enhanced biocompatibility, and the action of the M1 macrophages. Also, cellular viability and osteoblast activity increase in an implant surface, with a roughness between 1 and 100 μm [43]. Ma’s surface roughness was 0.871 ± 0.07 μm, which was slightly below the optimal range, while SLA’s roughness of 2.174 ± 0.07 μm was within the optimal range. The chemical components for Ma and SLA both had the majority composed of Ti and O. This suggests the formation of titanium oxide on the surface since Ti forms an oxide thickness of 3–5 nm at room temperature [44]. The oxide surface may promote the formation of an amorphous Ca compound, which can act as a biochemical link at the bone–implant interfaces [44]. The alumina particles on the SLA surfaces may have released into the surrounding tissues and interfered with the osseointegration, but the amount and effect were thought to be minimal.

In the same way, as in many other studies, rabbits were used to test the osseointegration and evaluate the healing of implants [27,45,46]. Woven bone to lamellar bone formation starts around 6–8 weeks and can take a few months to complete [36]. Few studies have concentrated on the biocompatibility of customized facial implant surfaces and materials. Facial implants differ from dental implants because they are not placed within the cancellous bone with all surfaces of the implant touching bone, but rather have only one surface lying on the cortical and cancellous bone. This position is unfavorable for osseointegration because it is less stable than endosseous dental implants and is dependent on screw stability and implant fit. In vivo experimental studies exploring the osseointegration of implants need a period of over 6 weeks to see the full healing response [28,36]. To maximize accuracy, surgical stents were manufactured, customized implants were used, three screws were fixated, and the periosteum was meticulously sutured. A protrusion on the lingual side of the implant helped to position and stabilize the implant. Surgical guides have proven to statistically improve the accuracy of implant placement in dentistry [47].

Pekkton^®^ ivory is a PEKK product enhanced with the addition of titanium dioxide, which increases the hardness and wear resistance and maximizes the esthetic appearance [48]. The same product was used in this study, and the EDS results showed elements of C, O, Ti, and Cl. Since titanium dioxide particles incorporate P and Ca ions into the surface layer and promote pre-osteoblastic activity, this may have been the reason for osteogenic differentiation [49,50,51]. Though the BIC value was smaller than that for titanium or SLA surfaces, PEKK is a promising material that warrants further study. In this study, there was a significant increase in the BIC from 8 to 12 weeks. Such an increase in the BIC value may be explained by the relatively slow infiltration of cells due to the minimally rough surface [52]. With the modification to the surface, so that it becomes porous, this delay is alleviated and the degree of osseointegration increased [49,53].

Ma, SLA, PEKK-surfaced implants and the periosteum

The periosteum plays an important role in bone formation because of the ample amount of neurovascular tissue and bone progenitor cells, as well as its ability to differentiate into osteoblasts [54]. A study by Lutz et al. [55] placed implants in the frontal bone of pigs, leaving the threads 5 and 10 mm above the cortical bone and covered by the periosteum. After 60 days, a significant vertical peri-implant bone was formed supracrestal, ranging from 6 to 36%. Cases of osseointegration and bone formation on the lateral side of porous hydroxyapatite scaffolds in the cranium after cranioplasty have also been reported [56]. In our study, new bone was formed on the lateral side of the implant below the periosteum. Such results were achieved since the periosteum covering the implant was not damaged. The periosteum was carefully identified during surgery, incised and elevated without tearing, and carefully repositioned during suturing. The facial implant may have pushed the periosteum to induce bone formation. In some cases, the new bone was directly attached to the surface of the implants, but in most cases, the newly formed bone was usually surrounded by soft tissue. This is a novel finding in facial implants, and long-term studies are needed to investigate how such reactions will impact the implant.

The etiology of skeletal changes in the aging mandible is either due to its natural process or the consequence of reduced function, or both. The problems associated with previous types of implants were that they would absorb the bone below the implant, form a fibrous capsule, or migrate from the initial position [2]. Osteoconduction may be increased by surface topography, pore size, and structure [53,57]. In this study, Ma, SLA, and PEKK implants using CAD/CAM techniques allowed for a precisely situated implant within bone. Increased surface porosity promotes osseointegration into the Ma, PEKK, and SLA surfaces. The BIC values of 50–80% are commonly seen with clinically successful implants [58]. Even though the SLA surfaces showed BIC levels of 44–50% in this study, this was still regarded as significant, since one surface was attached rather than surrounded by the dental implant. The elevated BIC levels of Ma and PEKK and the appearance of bone on the lateral periosteum also point to their potential to stimulate the functional load and prevent atrophy. Implanting a material that can act as a scaffold, such as a facial implant, between the bone and the periosteum may trigger osteoconduction [59]. Direct mechanical osseointegration of the implant onto an aging mandible may delay mandibular atrophy over the long term.

Limitations and recommendations

In this study, the increased BIC and new bone formation on both the medial and lateral surfaces of the implant indicate that Ma, SLA, and PEKK all have osteoconductive potential. However, this study only made a comparison between the 8- and 12-weeks, which was insufficient to prove the effectiveness of the facial implant on senile atrophy [60]. One limitation was that only the surface properties were investigated in this study. The mechanical stress due to the materials used on the mandible can lead to problems on bone tissue [61,62,63]. Another limitation is that, because two different surface samples were used on one side of the rabbit, the soft tissue reaction may have been influenced. To minimize any adverse effects, a gap between the facial implants was made when they were inserted, and biopsies of the soft tissue were obtained from those areas that were furthest from the gap between the two implants to minimize cross-reaction. This study did not have a sham operation which could be beneficial in future studies. Additional long-term studies with separate facial implants and a sham control should be conducted in the future.

## 5. Conclusions

The Ma, SLA, and PEKK facial implants are biocompatible, possessing osseointegration properties. SLA facial implants can enhance osseointegration and provoke minimal soft tissue reactions, reducing micromotion and making them the most suitable choice for implants. They provide an excellent environment for bone regeneration and, over the long term, may prevent atrophy caused by an aging mandible. The bone formation between the lateral surface of the facial implant and the periosteum may assist in the osseointegration and stabilization of the facial implant.

## Figures and Tables

**Table 1:** Evaluation of inflammation in histological sections of the different facial implant groups. Ma, SLA, and PEKK implant depending on percentage of inflammation in the soft tissue, field severity, granulation tissue formation, and level of soft tissue fibrosis. (n = number of samples).

		Ma		SLA		PEKK	
		8 w (*n* = 4)	12 w (*n* = 4)	8 w (*n* = 4)	12 w (*n* = 4)	8 w (*n* = 4)	12 w (*n* = 4)
Inflammation in whole tissue (%)	Average	20.63	25.63	14.25	12.63	5.88	15.75
Field severity (n)	Minimal	0	0	2	1	2	2
	Mild	3	2	1	2	2	2
	Moderate	1	1	0	0	0	0
	Severe	0	1	1	2	0	0
Granulation tissue formation (n)	Absent	3	3	4	4	4	3
	Present	1	1	0	0	0	1
Fibrosis in soft tissue (n)	Absent	0	0	0	0	0	0
	Loose, thin fiber	0	0	0	0	1	2
	Thick collagen, focal	2	3	2	1	1	2
	Thick collagen, diffuse	2	1	2	3	2	0

## Data Availability

The original contributions presented in the study are included in the article, further inquiries can be directed to the corresponding author.
